# Braun Enteroenterostomy Following Pancreaticoduodenectomy: A Systematic Review and Meta-Analysis: Erratum

**DOI:** 10.1097/01.md.0000484784.15522.08

**Published:** 2016-06-17

In the article “Braun Enteroenterostomy Following Pancreaticoduodenectomy: A Systematic Review and Meta-Analysis”,^1^ which appeared in Volume 94, Issue 32 of *Medicine*, reference citations in Tables 1–3 were originally incorrectly given. The article has since been corrected online.
